# Docking Studies and Biological Evaluation of a Potential β-Secretase Inhibitor of 3-Hydroxyhericenone F from *Hericium erinaceus*

**DOI:** 10.3389/fphar.2017.00219

**Published:** 2017-05-12

**Authors:** Chen Diling, Yong Tianqiao, Yang Jian, Zheng Chaoqun, Shuai Ou, Xie Yizhen

**Affiliations:** ^1^State Key Laboratory of Applied Microbiology Southern China, Guangdong Provincial Key Laboratory of Microbial Culture Collection and Application, Guangdong Open Laboratory of Applied Microbiology, Guangdong Institute of Microbiology Guangzhou, China; ^2^College of Chinese Material Medical, Guangzhou University of Chinese Medicine Guangzhou, China

**Keywords:** molecular docking, regulatory mechanism, *Hericium erinaceus*, active pharmaceutical ingredient, functional foods, 3-Hydroxyhericenone F, Alzheimer’s disease

## Abstract

Alzheimer’s disease (AD) is the most common neurodegenerative disorder, affecting approximately more than 5% of the population worldwide over the age 65, annually. The incidence of AD is expected to be higher in the next 10 years. AD patients experience poor prognosis and as a consequence new drugs and therapeutic strategies are required in order to improve the clinical responses and outcomes of AD. The purpose of the present study was to screen a certain number of potential compounds from herbal sources and investigate their corresponding mode of action. In the present study, the learning and memory effects of ethanol:water (8:2) extracts from *Hericium erinaceus* were evaluated on a dementia rat model. The model was established by intraperitoneal injection of 100 mg/kg/d D-galactose in rats. The results indicated that the extracts can significantly ameliorate the learning and memory abilities. Specific active ingredients were screened *in vivo* assays and the results were combined with molecular docking studies. Potential receptor–ligand interactions on the BACE1-inhibitor namely, 3-Hydroxyhericenone F (3HF) were investigated. The isolation of a limited amount of 3HF from the fruit body of *H. erinaceus* by chemical separation was conducted, and the mode of action of this compound was verified in NaN_3_-induced PC12 cells. The cell-based assays demonstrated that 3HF can significantly down-regulate the expression of BACE1 (*p* < 0.01), while additional AD intracellular markers namely, p-Tau and Aβ_1-42_ were further down-regulated (*p* < 0.05). The data further indicate that 3HF can ameliorate certain mitochondrial dysfunction conditions by the reversal of the decreasing level of mitochondrial respiratory chain complexes, the calcium ion levels ([Ca^2+^]), the inhibiton in the production of ROS, the increase in the mitochondrial membrane potential and ATP levels, and the regulation of the expression levels of the genes encoding for the p21, COX I, COX II, PARP1, and NF-κB proteins. The observations suggest the use of *H. erinaceus* in traditional medicine for the treatment of various neurological diseases and render 3HF as a promising naturally occurring chemical constituent for the treatment of AD via the inhibition of the β-secretase enzyme.

## Introduction

Alzheimer’s disease (AD) is the most common neurodegenerative disorder, affecting approximately more than 5% of the worldwide population over the age 65, annually. The incidence of the disease is expected to increase in the next 10 years. Despite significant advances in the therapeutic strategies developed for several diseases, AD remains an incurable disease to the majority of the population. AD patients experience poor prognosis and consequently new drugs and therapeutic strategies are required to improve the clinical response and outcome of the patients. AD is a chronic neurodegenerative disease that frequently exhibits a slow progression that is accompanied by a greater recession of the disease over time. The cause of AD is poorly understood, and among various biochemical and morphological events, the presence of neurofibrillary tangles, senile plaques, neuronal and synaptic loss is notably noted (Abbott, 2011). Mitochondrial dysfunction and an accumulation of reactive oxygen species promote redox imbalance and β or tau-induced neurotoxicity (Islam, 2017).

Several studies have documented that mitochondrial dysfunction is related to the aging process and to neurodegenerative disorders (Hirai et al., 2001; Calkins and Reddy, 2011; Swerdlow et al., 2013; Cha et al., 2015). Mitochondria play a central role in the production of ATP as a source of cellular energy via oxidative phosphorylation (Simpkins and Dykens, 2008). In addition, mitochondria exert central roles in apoptosis signaling, lipid synthesis, and intracellular calcium buffering. Mitochondrial functions are less efficient during brain aging and/or brain damage (Cho et al., 2010). Mitochondrial abnormalities have been identified in AD and related neurodegenerative disorders (Reddy, 2011). Aβ has been detected in mitochondria (Thal et al., 2013; Rijal Upadhaya et al., 2014) and has been reported to be transported into the intermembrane space of the mitochondria by the enzyme translocase of the outer mitochondria membrane (Chen and Yan, 2007; Hansson Petersen et al., 2008; Wang et al., 2009). Previous studies demonstrated that Aβ might disrupt mitochondrial function via inhibition of key enzymes in respiratory metabolism, such as α-ketoglutarate dehydrogenase, pyruvate dehydrogenase, and cytochrome oxidase (Casley et al., 2002; Crouch et al., 2005). In addition, the enzyme Aβ-binding alcohol dehydrogenase (ABAD) interacts with Aβ and induces Aβ-mediated toxicity in mitochondria, via the generation of ROS (Lustbader et al., 2004). Furthermore, over-expression of APP and/or exposure to Aβ have been shown to induce mitochondrial fragmentation and abnormal distribution, which result in mitochondrial and neuronal dysfunction (Barsoum et al., 2006; Rui et al., 2006; Wang et al., 2008). Certain studies have suggested dysfunction of mitochondria in Tauopathy patients and disease models (Bratic and Trifunovic, 2010; DuBoff et al., 2012), based on the reduced levels of the mitochondrial metabolic proteins including pyruvate dehydrogenase, ATP synthase, and Complex I (David et al., 2005; Rhein et al., 2009). The mitochondrion is the main source of ROS in the intracellular space, and the over-production of superoxide radical ($O_{2}^{•–}$) and hydroxyl radical (^-^OH), due to additional oxidative damage, causes an unrepaired damage to the mitochondrial DNA, thus leading to a functional defect in Complexes I and/or III (Islam, 2017). The aforementioned findings indicate that oxidative stress and mitochondrial dysfunction are associated with AD.

Amnesia is the loss of memory that is caused by hippocampal or medial temporal lobe damage and is a main clinical symptom of AD. During the past years, numerous studies have investigated the molecular mechanisms of learning/memory. As a reducing sugar, D-galactose is able to form advanced glycation end products (AGEs). Administration of D-galactose to human populations can induce cognitive deficits and disruptions in the synaptic communication. Thus, D-galactose-treated rats have been extensively used as a rodent model with synaptic disruption and memory impairment (Huang et al., 2016). The majority of the drugs, including some extracts from traditional Chinese medicines, have been tested for their ability to reverse D-galactose-induced memory deficits (Zhan et al., 2014; Gao et al., 2016; Li et al., 2016).

Sodium azide (NaN_3_) is a well-known COX inhibitor, which has been utilized over the last 20 years to induce metabolic compromise, resulting in an increase in amyloid production and the changes in the phosphorylation state of the tau protein (Selvatici et al., 2013; Delgado-Cortés et al., 2015). NaN_3_ has been further shown to cause memory deficit and neurodegeneration in rats (Szabados et al., 2004), and is a more potent oxidative stress inducer compared with H_2_O_2_ (Gao et al., 2007).

*Hericium erinaceus* belongs to the division of Basidiomycota and the class of Agaricomycetes and is an edible and medicinal mushroom. It is widespread across the continents as a delicacy and replaces pork and/or lamb in the Chinese vegetarian cuisine. *H. erinaceus* is rich in active constituents namely, diterpenoids, steroids, polysaccharides, and other functional ingredients that are used as natural plant resources. *H. erinaceus* exerts a plethora of biological effects namely, cognitive improvement (Mori et al., 2009), stimulation of nerve growth factors (Mori et al., 2008) and nerve cells (Wong et al., 2007) and hypoglycemic and anti-cancer effects (Kim et al., 2014; Li G. et al., 2014). Based on the aforementioned studies it was assumed that *H. erinaceus* may have potential activity on the treatment of AD, although no reports regarding the pharmacologically active ingredients and their corresponding mode of action have been documented. In the present study, the effects of ethanol:water (8:2) extracts from *H. erinaceus* were investigated on the amelioration of learning and memory processes in a D-galactose-induced deficit rat model. Subsequently, the specific active ingredients were screened by molecular docking studies, in order to identify novel biological components and novel modes of action for the treatment of AD. Furthermore, a NaN_3_-induced PC12 cell model was used to confirm the molecular docking studies.

## Materials and Methods

### Learning and Memory Effects Evaluation

#### D-Galactose-Induced Deficits Rats Prepared and Treatment

Adult male Sprague Dawley rats (180–220 g, obtained from Center of Laboratory Animal of Guangdong Province, SCXK [Yue] 2008-0020, SYXK [Yue] 2008-0085) were pair-housed in plastic cages in a temperature-controlled (25°C) colony room at a 12/12-h light/dark cycle. Food and water were available *ad libitum*. All experimental protocols were approved by the Center of Laboratory Animals of the Guangdong Institute of Microbiology. All efforts were made to minimize the number of animals used.

The rats were randomly divided into four groups as follows: control group that received oral distilled water, model group that received intraperitoneal injection (i.p) of 100 mg/kg/d D-galactose (Zhong et al., 2016; Liang et al., 2017), low-dose group that received i.p D-galactose (100 mg/kg/d), and gavage at a dosage of 50 mg/[kg⋅d] in ethanol:water (8:2) extracts from *H. erinaceus* (EH), and high-dose group that received D-galactose i.p (100 mg/kg/d) and gavage at a dosage of 100 mg/[kg⋅d] in ethanol:water (8:2) extracts from *H. erinaceus* (EH) every day in the morning, the dose of EH was according to other literatures (Zhang et al., 2016) proved that *H. erinaceus* extracts at doses of 0.3, 1.0, and 3.0 g/kg for 4 weeks can significantly enhanced the Ach and ChAT concentrations in serum and the hypothalamus in the AlCl_3_- and D-gal-induced AD mice. Every group consisted of eight animals and the procedure duration was 8 weeks.

#### Water Maze Tests

Rat spatial learning and memory abilities were tested in the Morris water maze (MWM, DMS-2, Chinese Academy of Medical Sciences Institute of Medicine). The MWM consisted of a circular opaque fiberglass pool (200-cm diameter) filled with water (25 ± 1°C). The pool was surrounded by light blue curtains, and three distal visual cues were fixed on the curtains. A total of four floor light sources of equal power provided uniform illumination to the pool and testing room. A CCD camera was placed above the center of the pool in order to record animal swim paths. The video output was digitized by an EthoVision tracking system (Noldus, Leesburg, VA, USA). The water maze tests included three periods: initial spatial training, spatial reversal training, the probe test and the procedures same to those described previously (Chen et al., 2014).

#### ADs’ Parameters Measurement

The appearance, behavior and the fur color of the animals were observed and documented every day. Animal weight was measured every 3 days during the drug administration period. Following the water maze testing, the blood and serum were acquired. Routine index and cytokines (Wang et al., 2016) were measured and the brains of the animals were dissected. A total of four brains from each group were fixed in 4% paraformaldehyde solution and prepared as paraffin sections. The sections were stained with hematoxylin-eosin (H&E) and immunohistochemistry staining and observed under light microscopy (Zeng et al., 2013; Chen et al., 2014).

### Molecular Docking of the Compounds Derived from *H. erinaceus* to the Human β-Secretase (β-site Amyloid Precursor Protein-Cleaving Enzyme 1; BACE1)

The pdb file regarding the crystal structure of human BACE1 with co-crystal ligand (*N*-[(1R)-1- (4- fluorophenyl) ethyl]-*N*′-[(2S,3S)-hydroxy-1-phenyl-4-(1H-pyrazol-1-yl)butan-2-yl]- 5-[methyl (methyl sulfonyl)amino] benzene -1,3-dicarboxamide; ZPX394) at the active binding site (3UQU.pdb) was downloaded from the RCSB protein data bank^1^ and prepared by deletion of the co-crystal ligand, water, urea, SO_4_^2-^ and Cl^-1^ molecules. Subsequently, hydrogen atoms were added to the model and the pH of the protein was adjusted to 7.4. For ligand preparation, the 2D structures of the compounds from *H. erinaceus* were sketched using ChemOffice 2004 and converted into 3D images using the prepare ligands module. The compounds were minimized with Smart Minimizer method using CHARMm force field in DS. The molecular docking procedure was carried out by site-features directed docking (LibDock) for the screen of potent candidates, while the investigation of receptor–ligand interactions was conducted using Discovery Studio 2.5 (DS2.5) according to previously published studies (Panek et al., 2017). The binding site of the protein was defined for all the atoms within 9 Å. The top 10 poses of the docking study were selected on the basis of LibDockScore rank. The interactions between the identified lead compounds and the BACE1 protein were analyzed.

### Verification Tests of the Molecular Docking Studies

#### Preparation of the 3HF

Dried material was loaded onto a column that was eluted with twofold greater volume of petroleum ether:ethanol (9:1, PE) for the volatile compounds and the fatty acid fraction. The samples were sequentially eluted with fourfold greater volume of ethanol:water (8:2) for the crude extraction and the elute was dried and loaded on the D-101 macroporous resin. The samples were subsequently eluted with twofold greater volume of petroleum ether and fourfold greater volume of acetone consecutively. The acetone elute was collected and evaporated to dryness. The extraction and separation process was developed in-house and the patent application is pending, and the extraction steps showed in **Figure 5A**.

#### Cell Culture and Treatment

PC12 cells were obtained from the American Type Culture Collection (Rockville, MD, USA). They were maintained in DMEM supplemented with 10% FBS at 37°C in a humidified atmosphere of 95% air and 5% CO_2_ and seeded in 25 cm^2^ culture dishes (Chen et al., 2013). The cells were initially stabilized at 37°C for 24 h, at 80% of confluence, and subsequently cultured in serum free medium following passaging. The cells were incubated with different concentrations of 3HF (final concentrations: 0.05, 1.00, and 1.50 μg/ml) for 2 h. NaN_3_ was incubated at a final concentration of 0.03 mM to the cell culture for an additional 24 h. All the cells used in the present study were undifferentiated.

#### Cell Viability Assay

The cell viability of PC12 following treatment of 3HF was measured by quantitative colorimetric assay using the cell counting kit-8 (CCK-8, Dojindo Laboratories, Japan) assay. The assay was conducted as described previously (Xiong et al., 2015). The cells were seeded in 96-well culture plates at a density of 2 × 10^4^ cells/well. Following drug treatment, 10 μL/well of CCK8 solution was added, and the cells were incubated at 37°C for 1 h. The optical density of each well was determined at 450 nm using a microplate reader (RT-2100C, USA). Cell viability was expressed as percentage of survival compared with the non-treated control. The data are presented as the mean and standard error of the mean (SEM) for three independent experiments.

#### Measurement of Intracellular ROS Production, Mitochondrial Membrane Potential, and [Ca^2+^]_i_

The intracellular ROS level was measured using the 2′, 7′-dichlorofluorescein diacetate (DCFH-DA) method (Chen et al., 2013), whereas the mitochondrial membrane potential was measured using rhodamine 123 fluorescent dye (Chen et al., 2013). The calcium ion concentration [Ca^2+^]_i_ was measured using the Fura-2/AM fluorescent dye (Chen et al., 2013). The results are expressed as percentage of activity compared with the non-treated control.

#### Measurement of Intracellular F_1_F_0_-ATPase, ATP, and NADH-CoQ Levels

The cells were seeded in 25 cm^2^ culture dishes. At 80% confluence, the cells incubated with different concentrations of 3HF (final concentrations: 0.05, 1.00, and 1.50 μg/ml) for 2 h. NaN_3_ was used at a final concentration of 0.03 mM and was subsequently added to the culture for an additional 24 h. At the end of the drug treatment, the cells were washed with D-Hanks solution, scraped from the plates in the presence of 1 mL ice-cold PBS (0.1 M, containing 0.05 mM EDTA) and homogenized. The homogenate was centrifuged at 4,000 × *g* for 10 min at 4°C. The resulting supernatants were stored at -80°C until further analyses. The activity of the enzymes F_1_F_0_-ATPase, NADH-CoQ reductase and the ATP levels within the mitochondria was determined using corresponding kits as demonstrated previously (McKenzie et al., 2007). The protein concentration of the cells was determined using the Coomassie Brilliant Blue G250 assay. The enzyme activities and protein content were all determined using the Detection kits purchased from Nanjing Jiancheng Bioengineering Insititute (Nanjing, Jiangsu, China). The procedures were carried out according to the manufacturer instruction. The levels were normalized to the protein concentration of each sample and expressed as a percentage of activity compared with the non-treated control.

#### Cell Mitochondrial Morphology

The presence of viable mitochondria was identified by Mito-tracker green (Molecular Probes, Beyotime, Shanghai, China) staining as described by Chang et al. (2014) with minor modifications. The stock solution of Mito-tracker green was prepared at a concentration of 1 mM in DMSO and stored at -20°C. The cells were stained in PBS with Mito-tracker Green (0.2 mM) at 37°C for 10 min. Following washing of the samples with PBS, the embryos were visualized using a fluorescent microscope at 490 nm.

#### Transmission Electron Microscopy (TEM)

The cells were fixed in 2.5% glutaraldehyde in PBS (pH 7.3). Following treatment with 1% osmium tetroxide solution (OsO_4_), 2% uranylacetate (UA) and dehydration in ethanol and acetone solvents, the samples were embedded in epoxy resin and polymerized for 48 h at 60°C. Ultrathin sections were cut using an ultramicrotome and placed on copper net (150 mesh). The sections on the grids were post-stained for 2 min with 1% UA, and for 6 min with 1% lead citrate by the addition of single drops of the staining solution at room temperature. The sections were rinsed in deionized water, dried and finally observed using a H7650 electron microscope (Hitachi, Tokyo, Japan), as described in previous studies (Ghobeh et al., 2014).

#### COX I, COX II, NF-κB, Caspase-3, and Caspase-9 Assays

Following drug treatment, the cells were washed with D-Hanks solution, scraped from the plates in the presence of 1 mL ice-cold PBS (0.1 M, containing 0.05 mM EDTA) and homogenized. The homogenate was centrifuged at 4,000 × *g* for 10 min at 4°C. The resulting supernatants were kept at -80°C until further analyses. The activities of COX I, COX II, NF-κB, Caspase-3, and Caspase-9 enzymes were measured by the Detection kits purchased from Cloud-Clone, Corp. (Houston, TX, USA). The procedures were carried out according to the manufacturer instruction. The levels of enzyme activity were normalized according to the protein concentration of each sample and expressed as a percentage of activity compared with non-treated control.

#### Preparation of Total RNA and Quantitative Reverse Transcriptase PCR (Q-RT PCR)

Cells were seeded in 25 mL plastic flask at a density of 2 × 10^5^ cells/mL and incubated with different concentrations of 3HF (final concentrations: 0.05, 1.00, and 1.50 μg/ml, respectively) for 2 h. NaN_3_ was used at a final concentration of 0.03 mM and was added to the culture for an additional 4, 8, or 24 h. The cells were harvested and the total RNA was extracted using Trizol reagent, whereas the remaining DNA was removed by DNase I. Consequently, purified RNA was obtained and the yield and purity was calculated by spectrophotometric estimation of the OD value at 260 and 280 nm respectively (k2800, Beijing Kai’ao Company).

A q-RT PCR assay was conducted using RNA obtained from PC12 cells. The expression levels of p21, PARP1, and NF-κB in mitochondria were examined. Amplification was carried out using ABI ViiA 7 Detection System and Hema9600 PCR (Zhuhai Heima medical equipment corporation) instruments. The mRNA levels of a control group were used as the baseline; As a result, ^ΔΔ^Ct was calculated using the formula ^ΔΔ^C_T_ = ^Δ^C_T_ of target gene -ΔC_T_ of the baseline. The fold change of the mRNA levels was calculated as fold = 2^-ΔΔCT^. The PCR primer sequences were as follows: sense: TCTTCTGCTGTGGGT CAGGAGG and antisense: GGCAGGCAGCGTATATCAGGAGA for p21;sense: TGCTGTCAAGGAGGA AGGTGTC and antisense: TCCAGGACGTGTGCAGAGTGTT for PARP1; sense: TGAGGAAGAGGCATG TAGAGACT and antisense: ACTGGCACTTCGGACAACAGAAG for NF-κB.

#### Western Blotting Analysis

Cells were seeded in 6-well culture plates at 5 × 10^6^ cells/well and were washed twice with D-Hanks solution following drug treatment. The cells were harvested and lysed with protein lysis buffer and the concentration of the protein was determined using the Coomassie Brilliant Blue G250 assay kit (Nanjing JianCheng Bioengineering Institute, China). Protein samples were separated by SDS-PAGE electrophoresis for 1.5 h at 90 V. The separated proteins were transferred to PVDF membranes using a transblotting apparatus (Bio-Rad Laboratories, USA) for 90 min at 90 V. The membranes were blocked with 5% (w/v) non-fat milk in TBS-T (Tris-buffer saline containing 0.1% Tween-20) at room temperature for 30 min and subsequently incubated at 4°C overnight with appropriate amount of primary antibody against BACE1 (ab183612), p-Tau (ab64193), Aβ_1-42_ (ab201060), PARP1 (ab191217), p21 (ab80633), and NF-κB p65 (ab16502) proteins. The primary antibodies were purchased from Abcam, Cambridge, UK, with the exception of the antibody for ARP1 (sc-393500) that was purchased from Santa Cruz Biotechnology, Inc. Following binding with the primary antibody, the membrane was washed with TBS-T once for 15 min and subsequently for 5 min for three times. The membrane was probed with horseradish peroxidase-conjugated secondary antibody at 4°C overnight. Incubation of the membranes with primary antibody against GAPDH at 4°C overnight, followed by a horseradish peroxidase-conjugated goat anti-mouse IgG at 37°C for 2 h was used as a loading control. The membrane were further washed with TBS-T for three times and the protein bands were visualized by ECL western blotting detection reagents (Amersham Biosciences, Buckinghamshire, UK).

### Statistical Analysis

Data are expressed as mean ± SD. Multiple group comparisons were conducted using one-way and two-way mixed analysis of variance (ANOVA) followed by Dunnett’s test in order to detect inter-group differences. A difference was considered statistically significant if the *p*-value was less than 0.05. Statistical Package for the Social Science (SPSS 17.0, SPSS Inc.) was used for statistical analysis in this study.

## Results

### Effects on Learning and Memory

#### Animal Appearance and Weight Control

**FIGURE 1:**
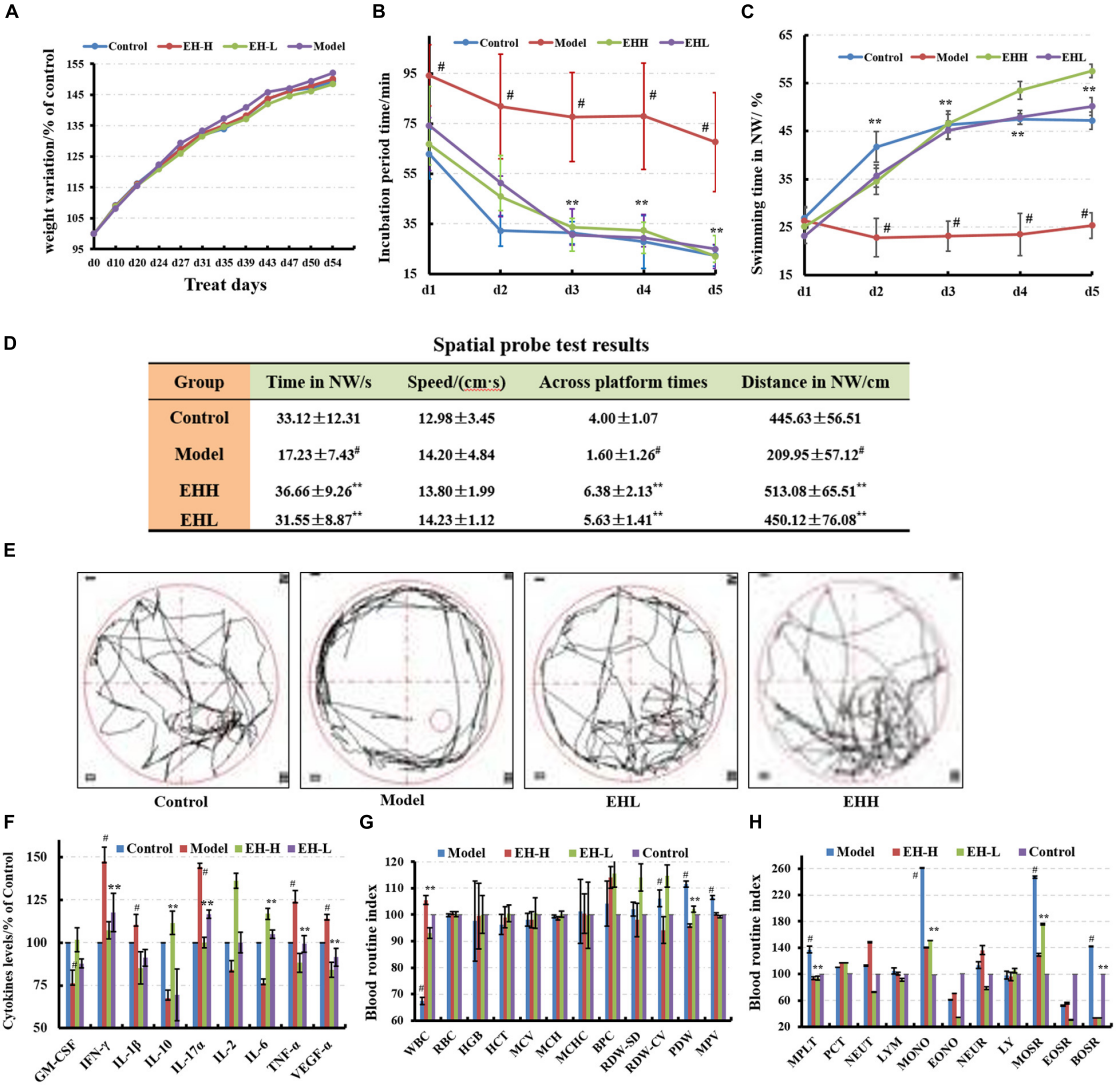
**Effects of ethanol:water (8:2) extracts from *Hericium erinaceus* (EH) on D-galactose-induced deficit in rats. (A)** Body weight changes during the treatment; **(B,C)** water maze tests results at the specified incubation periods and swimming times in NW; **(D)** spatial probe test results; **(E)** swimming trajectory during the spatial probe test; **(F)** cytokine levels of GM-CSF, TNF-γ, 1L-10, IL-2, 1L-17α, 1L-6, TNF-α, and VGEF-α in serum; **(G,H)** routine blood index changes. Control group (oral distilled water), model group [intraperitoneal injection (i.p) of 100 mg/kg/d D-galactose], low-dose group (concomitant administration by i.p injection of 100 mg/kg/d D-galactose and gavage at a dose of 50 mg/[kg⋅d] ethanol:water (8:2) extracts from *H. erinaceus* (EH)), high-dose group (concomitant administration by i.p injection of 100 mg/kg/d D-galactose and gavage at a dose of 50 mg/[kg⋅d] ethanol:water (8:2) extracts from *H. erinaceus* (EH)). Values are expressed as mean ± SD, ^#^*p* < 0.05 vs. control group, ^∗^*p* < 0.05, ^∗∗^*p* < 0.01 vs. model group, indicates significant differences compared with the model group.

The fur of the EH-treated animals was apparently smoother compared with that of the model group, which indicated that treatment with the EH can retain the fur at a healthy state. The weight records indicated that there were no significant difference (*F* = 2.55, *p* > 0.05) in the average weight change rate between the treated and the model groups, as shown in **Figure 1A**. The mean weight of the animals was approximately 330 ± 11.27 g at the beginning and 475 ± 20.00 g at the end of the experiment, which implied that the drugs exhibited no serious adverse effects on the animal weight.

#### Effects on Behavior

The incubation period for each EH-treated group (62.85 ± 11.97 s) was significantly shorter in length compared with that noted in the model group. The low-dose EH group incubation period was 74.16 ± 15.61 s, while the incubation period for the high-dose EH group was 66.73 ± 10.56 s on the first day. The differences noted in the incubation periods of the aforementioned groups were significant (*F* = 9.66, *p* < 0.05) compared with those noted in the model group (incubation period of 94.25 ± 12.20 s). On the fifth day, the low-dose EH group incubation period was 24.94 ± 5.30 s, whereas for the high-dose group the corresponding value was 21.98 ± 4.71 s. The differences were significant compared with the model group 67.65 ± 9.71 s (*F* = 98.71, *p* < 0.01), as shown in **Figure 1B**. Furthermore the swimming time in NW quadrant was improved (**Figure 1C**). The results indicated that EH can ameliorate D-galactose-induced learning and memory dysfunction in rats.

The probe test results indicated that there were no significant differences (*p* > 0.05) among the groups with regard to the total swimming distance and/or speed. The swimming time of the control group in the NW quadrant (33.12 ± 12.31 s) was longer compared with that noted in the other three quadrants, and the differences among them were significant (*F* = 6.63, *p* < 0.01). The swimming time of the model group was 17.23 ± 7.43 s, which was significantly lower compared with the control group (*p* < 0.01), suggesting that the rats couldn’t remember the location of the platform. The swimming times of the low- and high-dose EH groups were 31.55 ± 8.87 s, 36.66 ± 9.26 s in the NW quadrant, which were significantly greater in length compared with the model group (*F* = 6.63, *p* < 0.01). The differences noted in the parameters measured of the aforementioned groups were significant (*F* = 6.63, *p* < 0.01), as shown in **Figure 1D** compared with those noted in the model group. In addition, the swimming trajectory of the EH group was apparently denser in the NW quadrant compared with the other quadrants, as shown in **Figure 1E**. The results suggested that EH could ameliorate D-galactose-induced learning and memory dysfunction in rats.

#### Improvement on AD Parameters

##### Cytokines levels and blood routine index changes

The cytokine levels in the serum of the model group were significantly different from those noted in the control group (*p* < 0.05), and the levels of the pro-inflammatory cytokines increased, while the corresponding levels of the anti-inflammatory cytokines decreased (**Figure 1F**). Following treatment by EH, the cytokine levels were reversed to the levels noted in the control group, which indicated that treatment with the EH improved the inflammatory environmental factors. Certain blood biomarkers of the model group were apparently changed compared with the control group, while following treatment of EH, their levels were reversed to the levels noted in the control group (**Figures 1G,H**). This suggested that the EH can retain the D-galactose-induced deficit in the rat model.

##### Pathological and morphological findings

**FIGURE 2:**
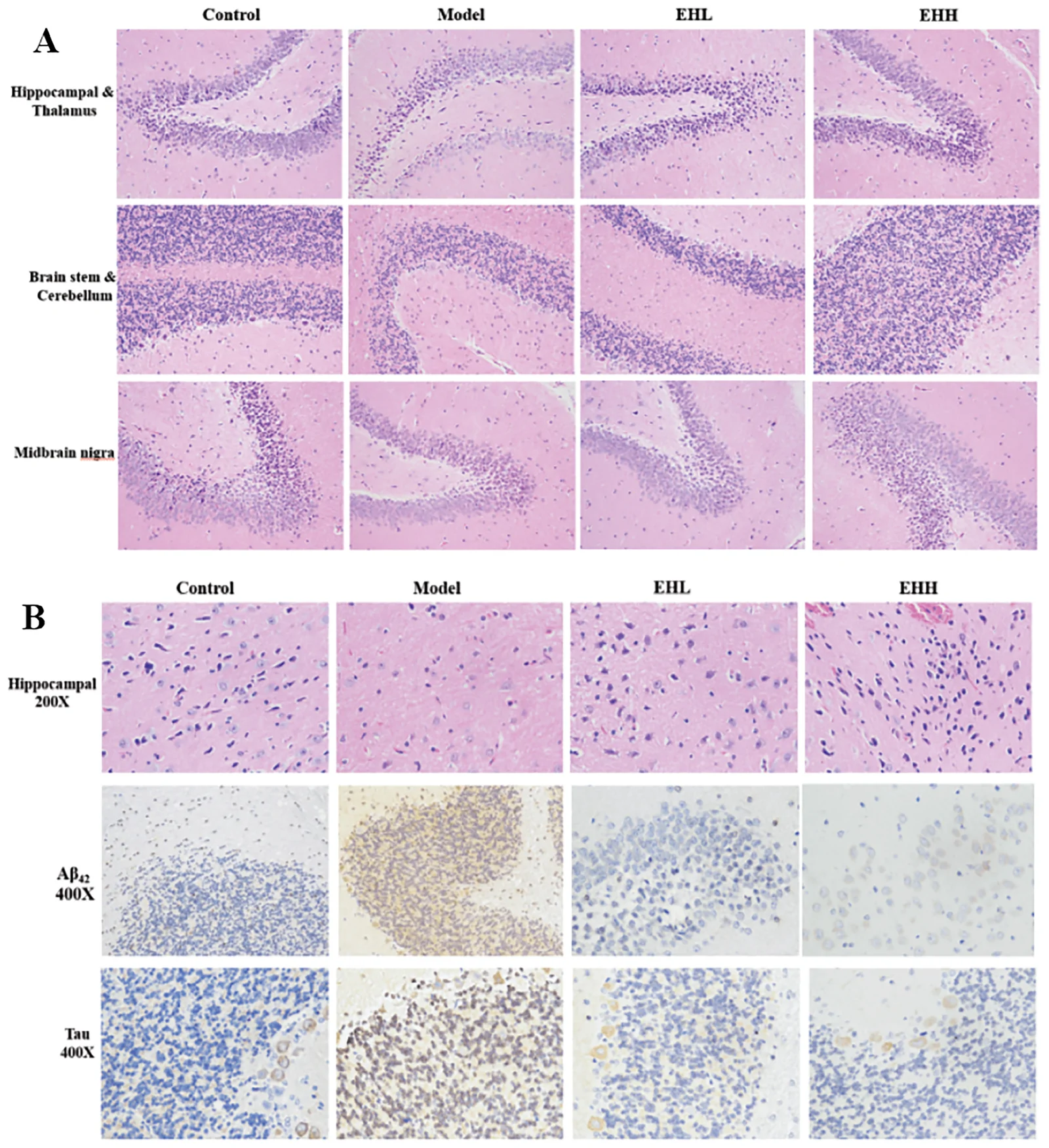
**Histopathological changes and immunohistochemistry staining of Tau and Aβ_42_ in the brain tissues of D-galactose- induced deficit rats. (A)** is the hematoxylin-eosin (HE) staining of hippocampal and thalamus, brain stem and cerebellum, midbrain nigra; **(B)** is HE staining of hippocampal, immunohistochemistry staining of the expression of Aβ_42_ and Tau protein in hippocampal.

Following hematoxylin-eosin (HE) staining, the histopathologic morphology of the model group exhibited apparent changes compared with the control group (**Figure 2A**), which indicated that the size of neurons in the cortex was smaller, and the cells were associated with nuclear pyknosis. The boundary of the cytoplasm and the nucleus was not distinct (**Figure 2B**). The cellular space between the neurons and the neurogliocytes had expanded, whereas the hippocampal pyramidal neuron had become smaller (**Figure 2B**). Following treatment with EH, the pathological changes noted in the model group were improved and the effect was proportional to the dose of administration.

Immunohistochemistry staining indicated that the expression of Tau (3.21 ± 0.17 fold of control) and Aβ_42_ (4.67 ± 0.28 fold of control) proteins in the brain tissues of D-galactose-induced rats was significantly increased compared with the control group (as onefold), while following treatment with EH, the changes noted were attenuated, especially the high dose group (0.69 ± 0.12 for Tau, 0.94 ± 0.11 for Aβ_42_). The pharmacological effect caused by EH treatment was proportional to the dose of administration.

### Molecular Docking of Compounds Extracted from *H. erinaceus* to BACE1 Enzyme and Investigation of the Receptor–Ligand Interactions

**FIGURE 3:**
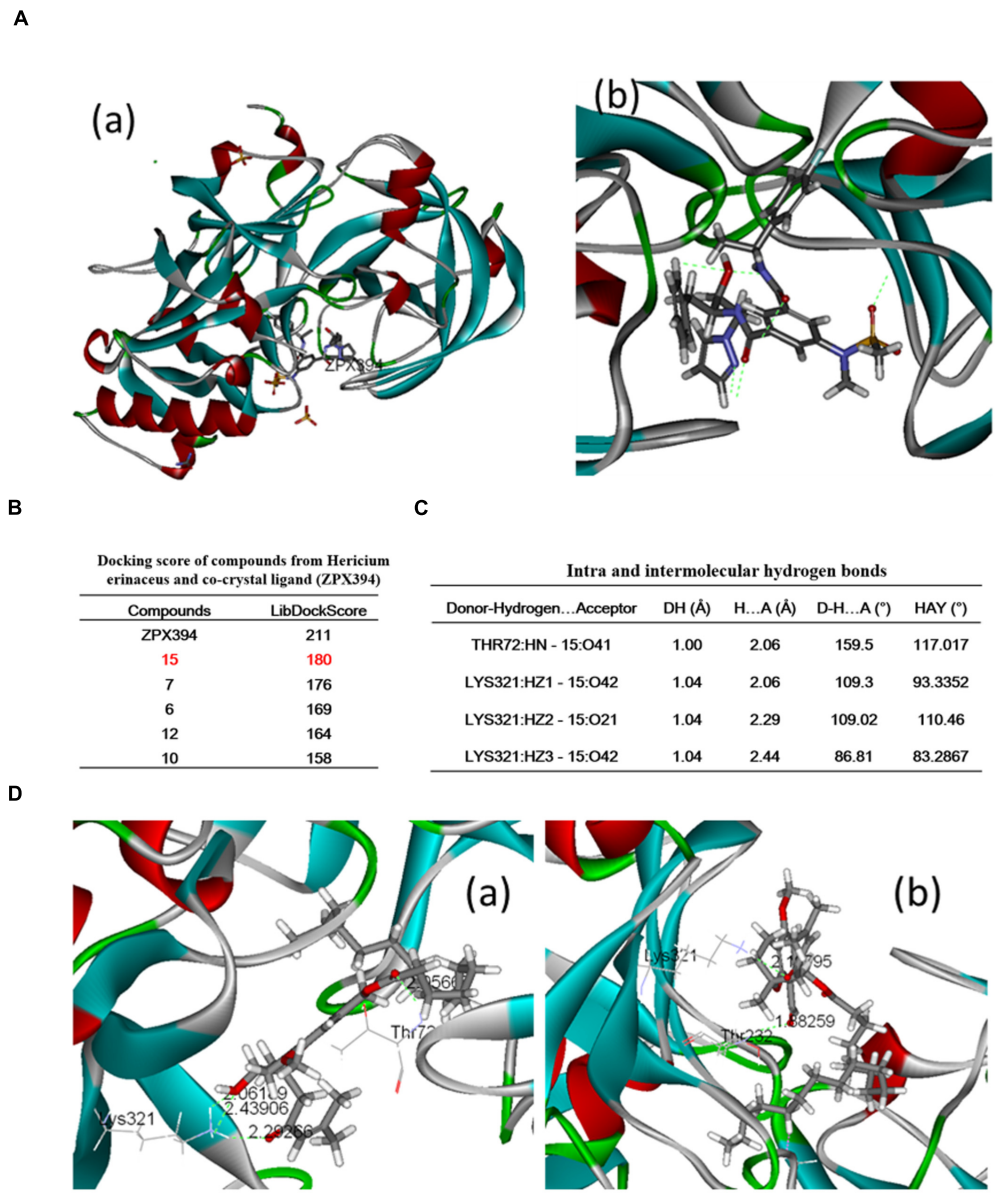
**Binding pattern of *H. erinaceus* compounds: (A-a)** crystal structure of BACE1; **(A-b)** binding modes and co-crystal ligand in BACE1; **(B)** docking scores of compounds from *H. erinaceus* and co-crystal ligand (ZPX394); **(C)** intra and intermolecular hydrogen bonds; **(D-a)** crystal structure of BACE1 and **(D-b)** 3-Hydroxyhericenone F-mediated hydrogen bond interactions with Lys321 and Thr72 (green line).

The molecular docking study was conducted using the LibDock protocol for the screen of potent candidates from *H. erinaceus* and the investigation of receptor–ligand interactions in the Discovery Studio 2.5 (DS2.5) software. A total of 17 components from *H. erinaceus* were collected from a literature search and 1048 poses were generated for all the compounds investigated. The docked poses were ranked by the LibDockScore and the top 10 poses with the co-crystal ligand for BACE1 were retained (**Figure 3B**). The data revealed four hits namely, compounds 15 (3HF), 7, 6, and 12 with corresponding scores of 180, 177, 169, and 164, respectively compared with the score noted for ZPX394 (211) (**Figure 3B**). This indicated that the four compounds identified may exert potent BACE1 inhibitory activity. The interaction between the BACE1 protein and the four compounds identified was further analyzed using Receptor–Ligand Interaction module in DS. The analysis between BACE1 and compound 15 (3HF) revealed that four hydrogen bond interactions appeared in the docked pose that were the following: the oxygen of the hydroxyl group was attached to the third position of the *O*-substituted hexyl ring, whereas the oxygen of the carbonyl group was attached on the second position of the 3-ene-4-methyl-2-pentone moiety of compound 15. This facilitated the interaction with the amine group of LYS321 with interference distance values of 2.062, 2.293, and 2.439 Å, respectively. The oxygen of the carbonyl group between the long alkyl group and the phenyl ring interacted with the amine group of Thr72 with interference distance value of 2.056 Å (**Figure 3C**). The interaction with LYS321 forms two 3-membered and 9-membered rings, which may stabilize the conformation. Furthermore, there is no bump between compound 15 and BACE1. This suggested optimal interaction between compound 15 and BACE1. Since compound 7 exhibited a high LibDock score (176), we further analyzed the interactions between compound 7 and the BACE1 protein. The oxygen of the carbonyl group was attached on the second position of the 3-ene-4-methyl-2-pentone moiety of the compound 7. This allowed an interaction with the amine group of LYS321 with interference distance value of 2.198 Å. Finally, an interaction of the amine group of Thr232 was observed with the oxygen atom of the carbonyl group attached on the phenyl ring of compound 7, with interference distance value of 1.883 (**Figure 3**).

### Verification of the Molecular Docking Studies

#### 3HF Significantly Inhibits the Expression of BACE1 in NaN_3_-Induced PC12 Cells

**FIGURE 4:**
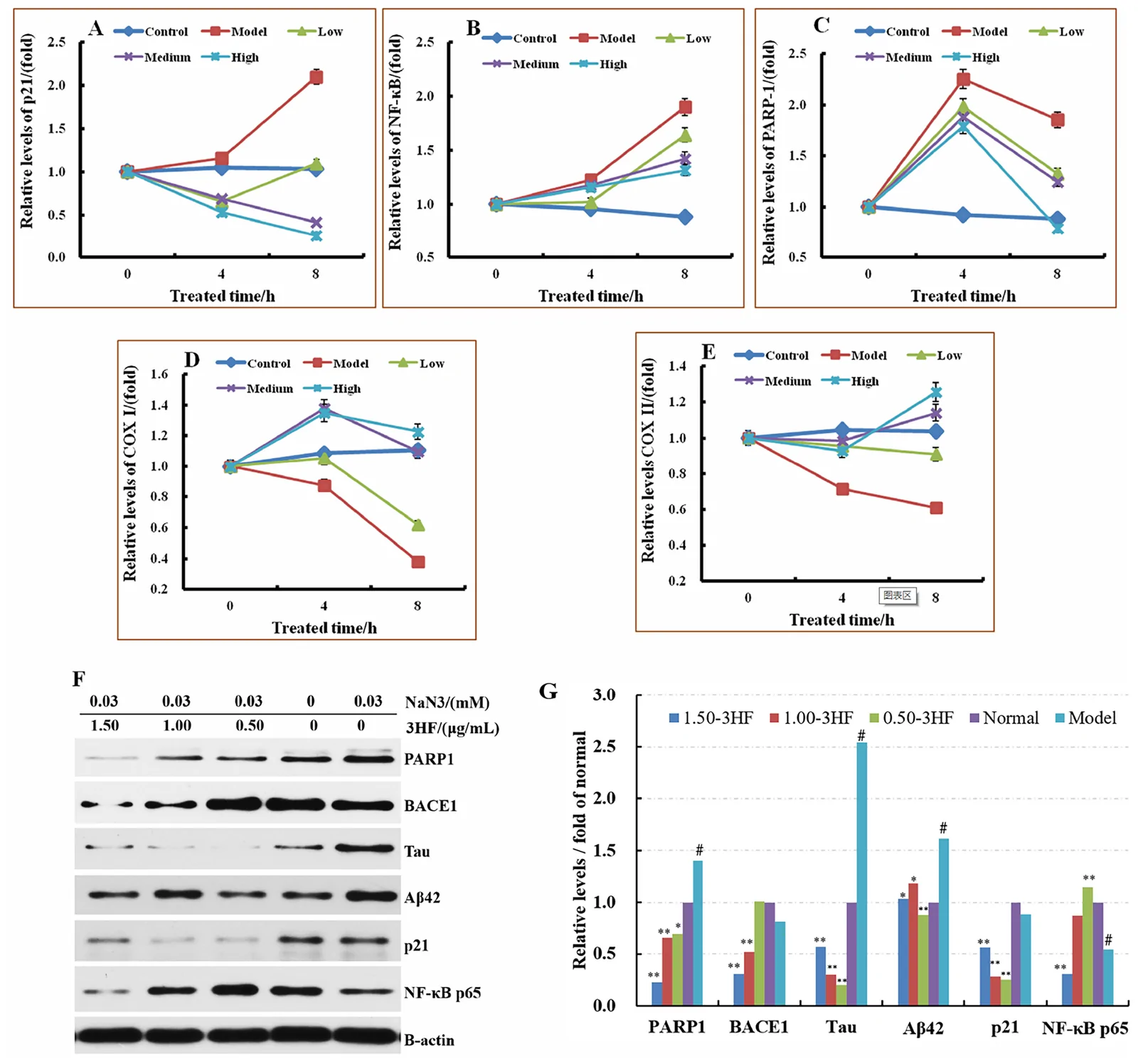
**Effects of 3HF on the mRNA expression levels of PARP1, p21, NF-κB p65, COX I, and COX II (A–E)** and the expression of the proteins related to mitochondrial dysfunction. 3HF reduced BACE1 although it reduced p-Tau and Aβ_42_ expression **(F,G)**. Cells were pretreated with 3HF at the concentrations of 0.05, 1.00, and 1.50 μg/ml for 2 h, followed by exposure to 0.03 mM of NaN_3_ for 12 h, and the protein extracts were used for Western blot analysis of the indicated proteins. The blots were probed with β-actin for the loading control validation ^#^*p* < 0.01 compared with control group; ^∗^*p* < 0.05 and ^∗∗^*p* < 0.01 compared with the NaN_3_-treated group.

The phosphorylation status of the intracellular markers that are involved in the neurodegenerative processes, such as tau, Aβ_1-42_ and beta-site amyloid precursor protein cleaving enzyme 1 (BACE1) was assessed by western immunoblotting. The expression of the modulators BACE1, p-Tau and Aβ_42_ was investigated in NaN_3_-induced PC12 cells. The cells were pretreated with 3HF at the concentrations of 0.05, 1.00, and 1.50 μg/ml for 2 h, followed by exposure to 0.03 mM of NaN_3_ for 4, 8, and 24 h (**Figure 4**). The expression of BACE1, p-Tau and Aβ_42_ proteins were decreased in all three 3HF treatment groups compared with the normal group. The expression of the proteins p21, NF-κB p65, and PARP1 that are related to mitochondrial dysfunction or cellular aging was further investigated. The data suggested that the expression levels of the aforementioned proteins were reversed following treatment with EH compared with the model group.

#### Effects of 3HF on the mRNA Expression Levels of Six Genes Related to Mitochondrial Dysfunction

The mRNA expression of six genes that are considered to be associated with the mitochondrial dysfunction processes was investigated in order to add further insight in the mechanism involved in the improvement of the mitochondrial dysfunction caused by 3HF (Picone et al., 2014; Ajith and Padmajanair, 2015). The cells were pretreated with 3HF at the concentrations of 0.5, 1.00, and 1.50 μg/ml for 2 h and further exposed to 0.03 mM of NaN_3_ for 4, and 8 h. The mRNA expression levels of p21, NF-κB p65, and PARP1 in the mitochondria were measured. The PARP1, p21, and NF-κB p65 expression levels increased following treatment from 4 to 8 h with 0.03 mM of NaN_3_. These increased trends were reversed by co-administration with different concentrations of 3HF, notably at the high 3HF dose (1.50 μg/ml) group, while the mRNA expression of the aforementioned genes was reversed to normal levels (**Figure 4**).

#### 3HF Improves the Cell Viability of NaN_3_-Induced PC12 Cells

**FIGURE 5:**
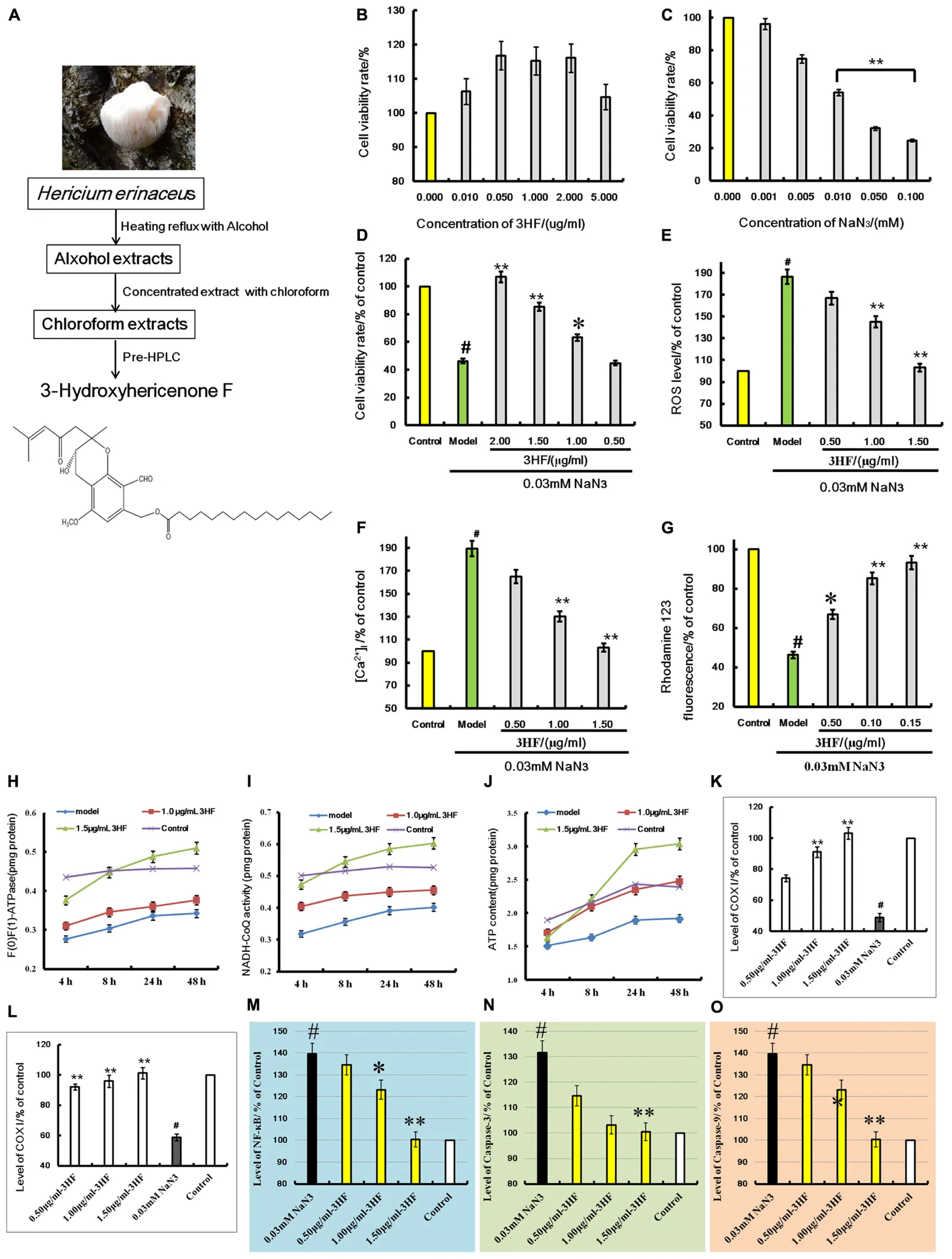
**(A)** is the extraction flowchart and chemical structure of 3HF. Effects of 3HF and NaN3 on PC12 cells **(B–D)** and effect of 3HF on ROS **(E)**, [Ca^2+^]_i_ **(F)**, mitochondrial membrane potential **(G)**, F_(1)_F_(0)_-ATPase **(H)**, NADH-CoQ **(I,J)**, COX I **(K)**, COX II **(L)**, NF-κB **(M)**, Caspase-3 **(N)**, and Caspase-9 **(O)** in NaN_3_-induced cytotoxicity PC12 cells. The cells were pretreated with 3HF at the concentrations of 0.05, 1.00 and 1.50 μg/ml for 2 h, followed by exposure to 0.03 mM of NaN_3_ for 24 h, whereas the control group was treated with PBS. Values are provided as the mean ± SD (*n* = 5) and % of the control group, ^#^*p* < 0.01 compared with control group; ^∗^*p* < 0.05 and ^∗∗^*p* < 0.01 compared with the NaN_3_-treated group, indicates significant differences compared with the model group.

Cell viability was assessed by the CCK-8 assay. PC12 cells were treated with different concentrations of 3HF for 24 h, and the cell viability was compared between 3HF-treated groups and control groups, showed in **Figure 5B**. When the cells were treated with 3HF at a concentration range of 0.01 to 2.00 μg/ml for 24 h, the cell viability was significantly increased compared with the control group (100%), while at the concentration of 5.00 μg/ml, the cell viability was decreased compared with the 2.00 μg/ml-treated group but increased compared with the control group. The results indicated that 3HF under 5.00 μg/ml was considerably non-toxic in PC12 cells.

Treatment of PC12 cells with a concentration range of 0 to 0.1 mM of NaN_3_ for 24 h induced cytotoxicity (**Figure 5C**). The cell viability was reduced to 24.74% of the control value (100%), as shown in **Figure 5B**. When the cells were co-incubated with 3HF at the concentrations of 0.05, 1.00, and 2.00 μg/ml for 2 h and further exposed to 0.03 mM of NaN_3_ for 24 h, the cell viability was significantly increased (63, 85, and 106% of the control value, respectively) compared with the NaN_3_ group (**Figure 5D**). The results indicated that 3HF conferred a protection against the NaN_3_-induced cytotoxicity in PC12 cells.

#### Effect of 3HF on NaN_3_-Induced Intracellular ROS, [Ca^2+^]_i_ and Mitochondrial Membrane Potential in PC12 Cells

Treatment of PC12 cells with 0.03 mM of NaN_3_ for 24 h caused a significant increase in the Calcium ion [Ca^2+^]_i_ levels (**Figure 5F**, 189.31% of the control value) with a concomitant decrease in the mitochondrial membrane potential (**Figure 5G**, 46.31% of the control value). When the cells were pretreated with 3HF at the concentrations of 0.05, 1.00, and 1.50 μg/ml for 2 h and further exposed to 0.03 mM of NaN_3_ for 24 h, the [Ca^2+^]_i_ levels were significantly reduced to 164.84, 130.26, and 103.16%, respectively, of the control value, while the mitochondrial membrane potential of the PC12 cells was significantly increased (66.84, 85.26, and 93.17% of the control value, respectively) compared with the NaN_3_ group. In addition, the ROS levels indicated a similar pattern of change with that noted in the [Ca^2+^]_i_ levels (**Figure 5E**).

#### Influence of 3HF on the Activities of ATP, NADH-CoQ, and F_(1)_F_(0)_-ATPase in the Mitochondria of the PC12 Cells

The mitochondria complexes I, V and ATP levels were markedly increased in the 1.00 and 1.50 μg/ml 3HF treated-groups, while the latter treatment of 3HF slightly increased complex I activity and ATP levels compared with the model groups (**Figures 5H–J**).

#### Effects of 3HF on the Expression of COX I, COX II, NF-κB, Caspase-3, and Caspase-9 Proteins

The activities of COX I, COX II, NF-κB, Caspase-3, and Caspase-9 proteins were detected using an ELISA method in order to clarify the protective mechanism of 3HF on the NaN_3_-induced PC12 cells. The levels of COX I (**Figure 5K**) and COX II (**Figure 5L**) were significantly reduced when the cells were treated with 0.03 mM NaN_3_ for 24 h, while the NF-κB (**Figure 5M**), caspase-3 (**Figure 5N**), and caspase-9 (**Figure 5O**) levels were significantly reduced. Following pretreatment of the cells with 3HF at the concentrations of 0.05, 1.00, and 1.50 μg/ml for 2 h and subsequent exposure to 0.03 mM of NaN_3_, the protein (COX I, COX II, NF-κB, Caspase-3, and Caspase-9) expression was approximately reversed to the normal levels.

#### Cell Mitochondria Morphology

**FIGURE 6:**
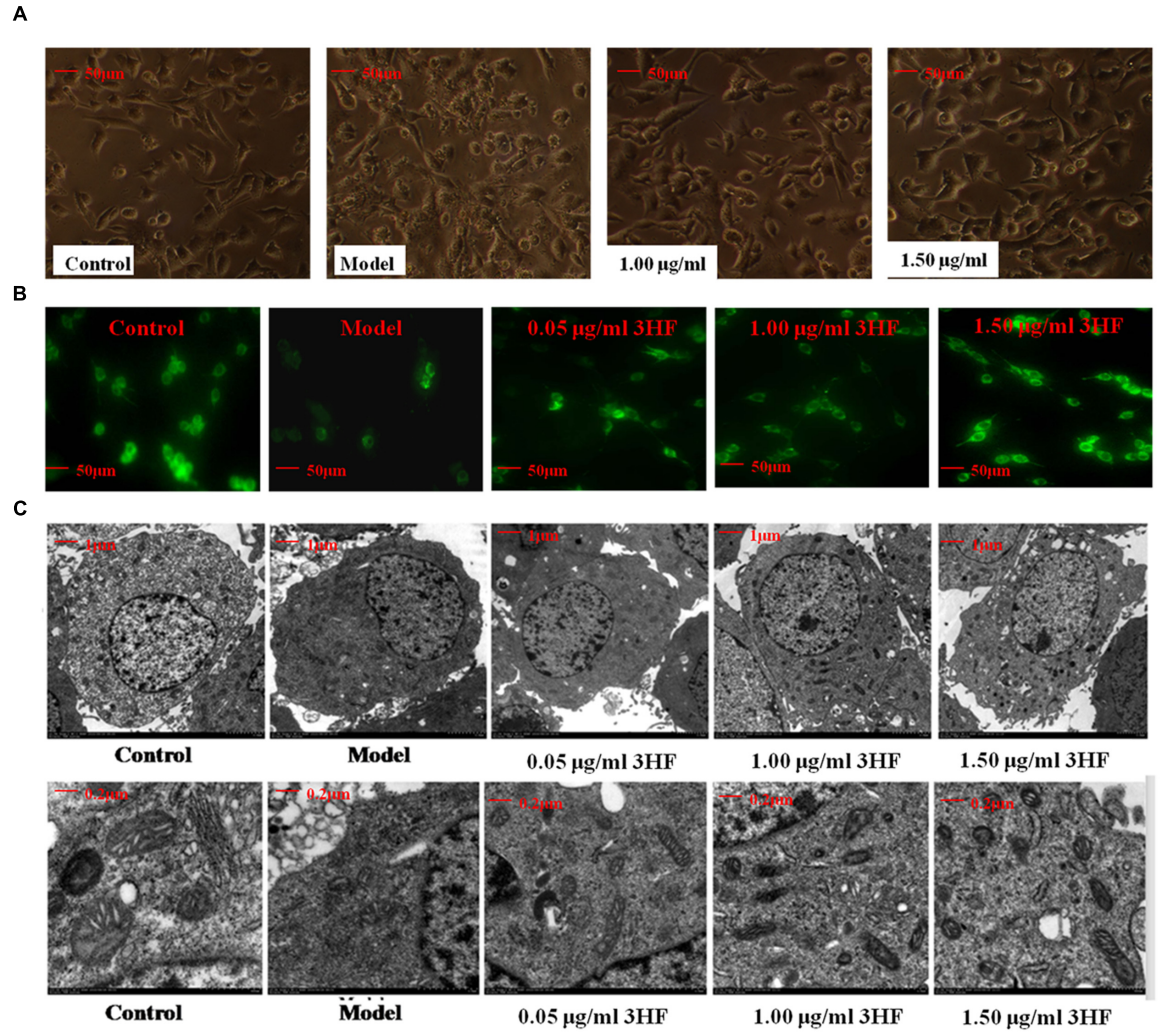
**Effect of 3HF on the cellular and mitochondrial morphology. (A)** The investigation was conducted by inverted microscope; **(B)** The investigation was conducted by fluorescent microscope at the wavelength of 490 nm. The number of cells and fluorescence intensity of the model group (treated with 0.03 mM NaN_3_ for 24 h) were lower compared with the control, while the 3HF increased the number of cells and fluorescence intensity; **(C)** The analysis was carried out by electron microscopy (1 and 5K). The 0.03 mM NaN_3_ treated groups indicated an ultrastructural change with disappearance of the nucleolus, condensed chromatin that was localized to the inner side of an intact nuclear membrane, blur mitochondria with disintegration and substantial lysis of cristae in PC12 cells.

The morphology of the mitochondria was evaluated by microscopy (phase-contrast, fluorescence, and electron microscopy) following treatment of PC12 cells by 0.03 mM of NaN_3_. The morphological assessment revealed swelling, shrinking cells and lower fluorescence intensity (38.19% of control) compared with the control samples, while in the 3HF-treated groups these effects were attenuated (**Figures 6A,B**), the fluorescence intensities were increased to 43.15, 61.27, and 86.78% of the control value, respectively. Furthermore, we used electron microscopy to determine the ultrastructural changes in the cells following NaN_3_ treatment. The data indicated that the nucleolus disappeared and the condensed chromatin was localized to the inner side of an intact nuclear membrane (**Figure 6C**). The mitochondria exhibited blur appearance and the boundary was not clear with concomitant disintegration and lysis of the cristae in the NaN_3_-treated PC12 cells compared with untreated control cells (**Figure 6C**). Based on the aforementioned observation, it was concluded that 3HF significantly alleviated the NaN_3_-induced ultrastructural changes that were characteristic of cellular damage (**Figure 6C**).

## Discussion

Various food constituents have been proposed as disease-modifying agents for AD, due to epidemiological evidence of their beneficial effects, and due to their ability to ameliorate the factors that are associated with the pathogenesis of AD pathogenesis (Chan et al., 2016). Such constituents have been demonstrated to bind iron, copper and zinc, scavenge reactive oxygen species and suppress the fibrillation of amyloid-beta peptide (Aβ), thus contributing to the prevention of AD (Chan et al., 2016). Mushrooms were originally used as a nutritional supplement, although in certain cultures, they have been traditionally exploited for their potential as medicinal remedies for various diseases. A majority of medicinal mushrooms were reported to affect the nervous system namely, *Lion’s Mane, H. erinaceus* and *Tiger Milk, Lignosus rhinocerotis*. The latter two mushrooms can stimulate neurite outgrowth (Samberkar et al., 2015), whereas *Phellinus igniarius* can reduce transient cerebral ischemia-induced neuronal death, suppress oxidative injury and disrupt the blood–brain barrier via microglia activation (Kim et al., 2015). *Agaricus blazei* protects the brain against oxidative stress-induced damage and increases mitochondrial-coupled respiration (de Sá-Nakanishi et al., 2014). *Hericium ramosum* mycelia extracts exert antioxidant activity and NGF synthetic activity. It has been shown that Dictyoquinazols extracted from *Dictyophora indusiata* protect primary cultured mouse cortical neurons from glutamate-and NMDA-induced excitotoxicities (Lee et al., 2002). Furthermore, the methanolic extract of *Chaga* enhances the cognitive and anti-oxidant activities of scopolamine-induced experimental amnesia (Giridharan et al., 2011). In addition, the oligosaccharide fraction isolated from the mycelium of the Lingzhi and/or Reishi medicinal mushroom *Ganoderma lucidum* exhibits anticonvulsant and neuroprotective effects (Tello et al., 2013). The chemical constituents from *H. erinaceus* can stimulate NGF-mediated neurite outgrowth (Phan et al., 2013; Zhang et al., 2015), while Erinacine A can act as an anti-neuroinflammatory agent that confers neuroprotection in Parkinson’s disease rat model (Kuo et al., 2016). The same compound can ameliorate AD-related pathologies in APPswe/PS1dE9 transgenic mice (Tsai-Teng et al., 2016). Moreover, the polysaccharides extracted from *H. erinaceus* exhibit antioxidant and neuroprotective effects on Aβ-induced neurotoxicity in neurons (Cheng et al., 2016), and are considered a potent immunostimulant for murine bone marrow-derived dendritic cell maturation (Qin et al., 2017). *H. erinaceus* has been shown to delay the onset of age-associated neurodegenerative diseases (Phan et al., 2014; Trovato et al., 2016), and increase mossy fiber-CA3 hippocampal neurotransmission and recognition memory in wild-type mice, when administered as a dietary supplement (Brandalise et al., 2017). Finally, *H. erinaceus* extracts can alter the behavioral rhythm in mice (Furuta et al., 2016) and possess neuroprotective properties in glutamate-damaged differentiated PC12 cells and in an AD mouse model (Zhang et al., 2016). Based on the aforementioned studies regarding the neuronal effects of medicinal mushrooms, *H. erinaceus* was selected in the present study in order to examine further the pharmacologically active ingredients and corresponding mode of action with regard to the protection against AD. The memory deficit rats were prepared by intraperitoneal injection (i.p) of 100 mg/kg/d D-galactose daily for a total period of 8 weeks, and the water maze tests were used to evaluate the improvement of learning and memory activities. The data indicated that the learning and memory activities were impaired compared with those of the control group (*p* < 0.05), while treatment with the EH could reverse the memory deficits. This finding indicated the identification of certain active ingredients with regard to the treatment of AD in the fruit bodies of *H. erinaceus*.

Beta-site amyloid-β protein precursor cleaving enzyme 1 (BACE1) is the rate limiting enzyme and the initial protease involved in the biosynthetic pathway of amyloid-β (Aβ). BACE1 is considered a potential disease-modifying target for the development of therapeutic drugs for AD (Devraj et al., 2016; Evin, 2016). The research of our group is focused on the screening evaluation of potent BACE1 inhibitors in an effort to identify suitable AD drug candidates. A molecular docking analysis was carried out in order to evaluate the BACE1 inhibitory effect of the compounds from *H. erinaceus*. The molecular docking study was conducted using the LibDock protocol in order to identify potent candidates and investigate receptor–ligand interactions in the Discovery Studio 2.5 (DS2.5) software. The data indicated that the docking score of compounds derived from *H. erinaceus* and the co-crystal ligands were as follows: 211, 180, 176, 169, 164, and 154 for ZPX394, 3-Hydroxyhericenone F (15), Hericenone G (7), Hericenone F (6), Hericerin (12), and Hericene B, respectively. This suggested four compounds with potential activity against BACE1 inhibition. It is tempting to speculate that some of these compounds may the active ingredients of *H. erinaceus* that have demonstrated potent activity for the treatment of AD, although further assays are required to be conducted for such a hypothesis notably for the remaining compounds from *H. erinaceus*, such as compound 7.

Mitochondria are considered important contributors to the development of several aging-associated diseases via the production of reactive oxygen species. The mitochondrial free-radical theory suggests that the progressive alteration of mitochondria that occurs during the aging process results in the increased production of ROS that in turn causes further mitochondrial dysfunction and damage to the cell (López-Otín et al., 2013). The most common paradigm of the failure in mitochondrial electron transport chain (ETC) enzymes reported in AD is the cytochrome c oxidase (COX, complex IV). The activity of cytochrome c oxidase is deficient in different brain regions, in particular in the cerebral cortex and hippocampus (Parker et al., 1994a,b; Fukui et al., 2007). Certain studies further demonstrated that COX inhibition leads to tau hyperphosphorylation and Aβ deposition, whereas the activity of COX is inhibited by the increased levels of Aβ that eventually results in cell death (Cassarino and Bennett, 1999; Eckert et al., 2010; Silva et al., 2011). In the present study, the levels of intracellular ROS were apparently induced, following treatment of 0.03 mM NaN_3_ for 24 h, which resulted in a change in the mitochondrial membrane potential and calcium ions ([Ca^2+^]_i_) influx. As a consequence, mitochondrial dysfunction occurred. When the cells were pretreated with 3HF at the concentrations of 0.05, 1.00, and 1.50 μg/ml for 2 h and subsequently exposed to 0.03 mM of NaN_3_, the aforementioned changes returned to normal levels, which indicated that 3HF can reverse and/or alleviate the NaN_3_-induced oxidative damage in PC12 cells.

Calcium ions (Ca^2+^) play an important role in normal neurotransmission, long- and short-term plasticity, and regulation of gene transcription. The disturbance in Ca^2+^ homeostasis potentiates excitotoxiciticy. Previous studies have demonstrated that the intracellular Ca^2+^ overload promotes cytochrome C release from the mitochondria, via the activation of the NF-κB pathway and the induction of caspase-9 and caspase-3 expression. These processes ultimately lead to cellular apoptosis (Chen et al., 2013; Li J. et al., 2014; Shi et al., 2014). In the present study, 3HF was shown to reduce Ca^2+^ overload and reversed the levels of p21, NF-κB p65, Caspase-9, Caspase-3, and PARP1 proteins. The latter findings demonstrated that 3HF inhibited cell apoptosis via the calcium channel.

Oxidative stress plays a major role in the development of neurodegenerative disorders (Islam, 2017). The age and various genetic and environmental risk factors cause an imbalance in the oxidative-redox system, while the levels of ROS are increased, which in turn stimulates pro-inflammatory gene transcription and release of cytokines and chemokines, such as IL-1, IL-6, and TNF-α. This eventually leads to the neuroinflammatory processes (Prasad, 2016). Chronic neuroinflammation is responsible for the loss of neurons. The levels of the cytokines IFN-γ, IL-1β, IL-17α, and TNF-α in the serum (**Figure 1B**) of D-galactose-induced rats were increased compared with those of control rats, while administration with EH decreased the cytokine levels. The extent of reduction correlated to the doses, whereas EH further decreased the production of ROS in NaN_3_-induced oxidative damaged cells, which indicated that it can improve the inflammation environment via the regulation of ROS levels.

Hyperphosphorylation of tau is involved in the formation of neurofibrillar tangles and is a central biochemical event in the pathogenesis of AD (Lee et al., 2001). Tau is a phosphoprotein with 80 potential serine/threonine and five tyrosine phosphorylation sites (Hanger et al., 2009; Wang et al., 2013). A number of sites, including Ser^396/404^, have been associated with neurons in the “pre-tangle” stadium in the brains derived from AD subjects (Bonda et al., 2011; Vingtdeux et al., 2011; Hu et al., 2013). Over-expression of p25 that results from calpain cleavage of p35, has been suggested to be involved in the hyperphosphorylation of tau protein (Camins et al., 2006) and in the formation of Aβ in AD (Liu et al., 2003), possibly coordinating the action of BACE1. BACE1 levels are raised in AD and potentially accelerate the initiating event for the production of Aβ (Liang et al., 2010). Previous studies have demonstrated that hericenones isolated from the fruit body of *H. erinaceus* promoted NGF biosynthesis in rodent cultured astrocytes (Kawagishia et al., 1991; Ma et al., 2010). The results reported in the present study are in agreement with these findings and the compounds in *H. erinaceus* were identified as promising naturally occurring chemical constituents worthy of further development for the pharmaceutical therapy of AD. In the current study, the expression of BACE1 and p-Tau were decreased following treatment with different concentrations of 3HF in NaN_3_-induced oxidative damaged cells (**Figure 4**), whereas EH further decreased the expression of tau and Aβ_42_ in the brain of the D-galactose-induced rat model (**Figure 2**).

The present study provides considerable information regarding the use of *H. erinaceus* for the treatment of various neurological diseases, and highlights 3HF as a promising naturally occurring chemical constituent for the therapeutic intervention of AD via the inhibition of the enzyme β-secretase. Although further studies are required to fully validate the efficacy of this compound in the treatment of AD, the data reported indicate that 3HF can ameliorate neuronal damage by reversing the decreased levels of [Ca^2+^]_i_ and ROS and improve mitochondrial function, via the increase in mitochondrial membrane potential and ATP levels of the mitochondrial respiratory chain complexes. The aforementioned processes result in decreased expression levels of the AD intracellular markers BACE1, p-Tau, and Aβ_42_.

## Author Contributions

CD, YT, YJ, ZC, SO, and XY: conceived and designed the experiments. CD, ZC, YJ, and SO: performed the experiments. CD, ZC, YT, and YJ: analyzed the data. CD and YT: wrote the paper and edited the manuscript. All authors read and approved the final manuscript.

## Conflict of Interest Statement

The authors declare that the research was conducted in the absence of any commercial or financial relationships that could be construed as a potential conflict of interest.

## Footnotes

1
http://www.pdb.org
