# Correction: Docking studies and biological evaluation of a potential β-secretase inhibitor of 3-Hydroxyhericenone F from *Hericium erinaceus*

**DOI:** 10.3389/fphar.2026.1943698

**Published:** 2026-08-06

**Authors:** Chen Diling, Yong Tianqiao, Yang Jian, Zheng Chaoqun, Shuai Ou, Xie Yizhen

**Affiliations:** 1 State Key Laboratory of Applied Microbiology Southern China, Guangdong Provincial Key Laboratory of Microbial Culture Collection and Application, Guangdong Open Laboratory of Applied Microbiology, Guangdong Institute of Microbiology, Guangzhou, China; 2 College of Chinese Material Medical, Guangzhou University of Chinese Medicine, Guangzhou, China

**Keywords:** molecular docking, regulatory mechanism, *Hericium erinaceus*, active pharmaceutical ingredient, functional foods, 3-Hydroxyhericenone F, Alzheimer’s disease

There was a mistake in Figures 2, 4 as published. The IHC images of Aβ_42_ in EHH in Figure 2B were wrong and the bands of Tau and Aβ_42_ in Figure 4F were misused. The corrected Figures 2, 4 appear below.

**FIGURE 2:**
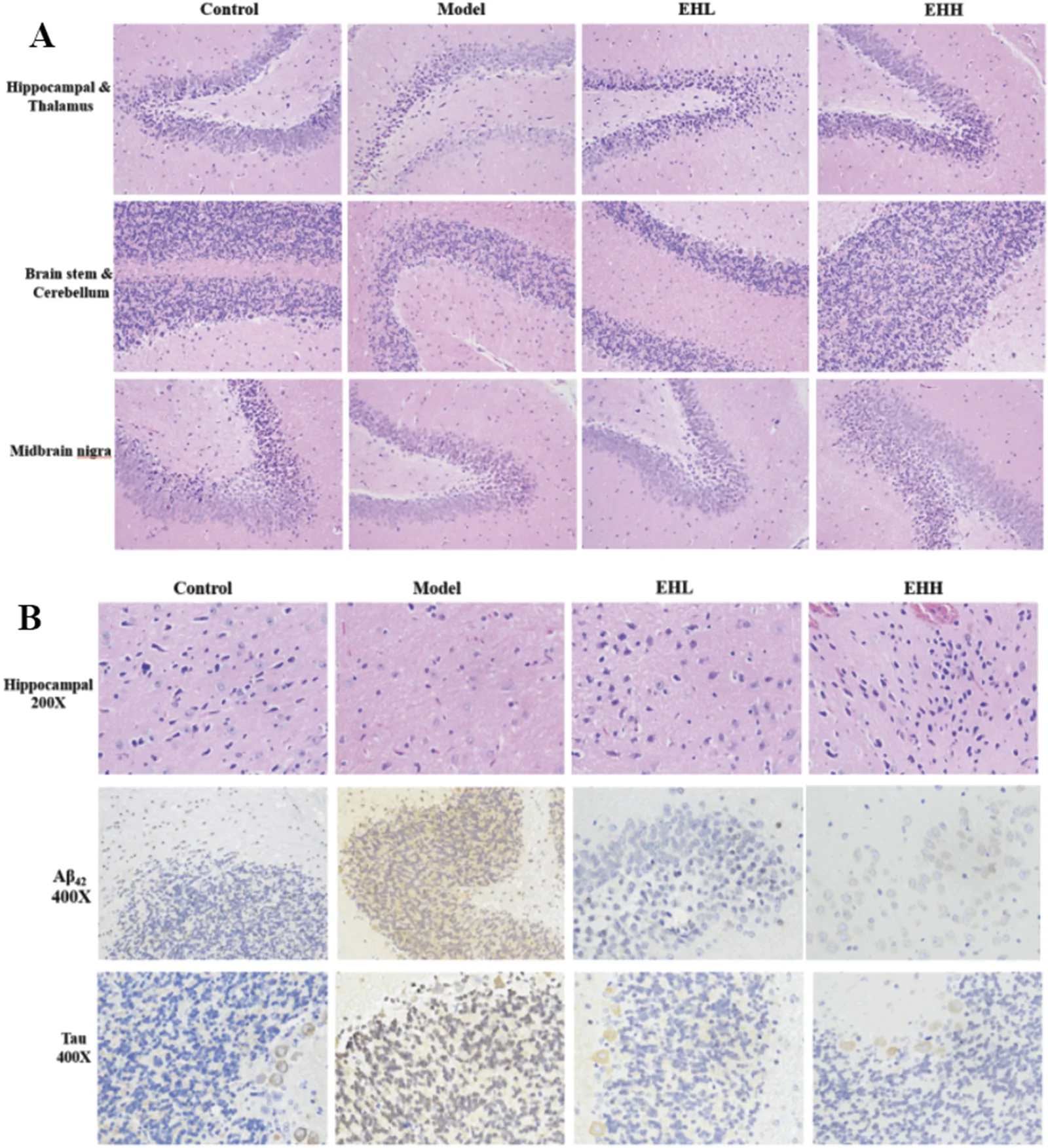
Histopathological changes and immunohistochemistry staining of Tau and Aβ_42_ in the brain tissues of D-galactose- induced deficit rats. **(A)** is the hematoxylin-eosin (HE) staining of hippocampal and thalamus, brain stem and cerebellum, midbrain nigra; **(B)** is HE staining of hippocampal, immunohistochemistry staining of the expression of Aβ_42_ and Tau protein in hippocampal.

**FIGURE 4:**
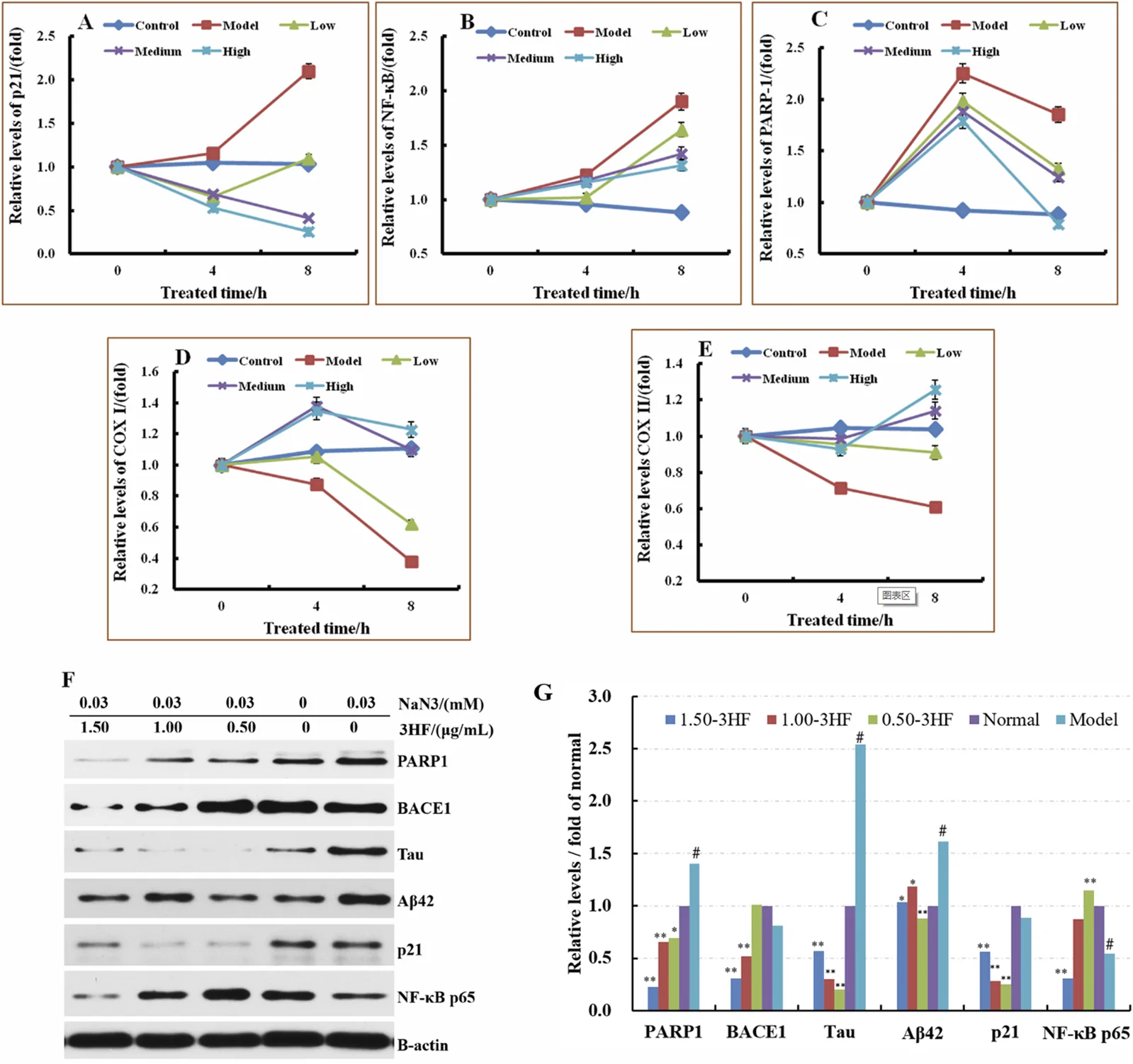
Effects of 3HF on the mRNA expression levels of PARP1, p21, NF-kB p65, COX I, and COX II **(A–E)** and the expression of the proteins related to mitochondrial dysfunction. 3HF reduced BACE1 although it reduced p-Tau and Aβ_42_ expression **(F,G)**. Cells were pretreated with 3HF at the concentrations of 0.05, 1.00, and 1.50 μg/mL for 2 h, followed by exposure to 0.03 mM of NaN_3_ for 12 h, and the protein extracts were used for Western blot analysis of the indicated proteins. The blots were probed with b-actin for the loading control validation ^#^ *p* < 0.01 compared with Normal (Control) group; *p < 0.05 and **p < 0.01 compared with the Model (NaN_3_-treated) group.

The original version of this article has been updated.

